# Developing a nomogram for predicting acute complicated course in pediatric acute hematogenous osteomyelitis

**DOI:** 10.1186/s13052-024-01703-z

**Published:** 2024-07-29

**Authors:** Chaochen Zhao, Qizhi Jiang, Wangqiang Wu, Yiming Shen, Yujie Zhu, Xiaodong Wang

**Affiliations:** 1 Department of Orthopedics, Shanghai Children’s Hospital, School of Medicine, Shanghai Jiao Tong University, Shanghai, China; 2 Department of Orthopaedics, Children’s Hospital of Soochow University, Suzhou, Jiangsu Province China

**Keywords:** Acute hematogenous osteomyelitis, Child, Predict, Complication, Nomogram, Biomarkers

## Abstract

**Background:**

The objective of this study was to develop and validate a nomogram for predicting the risk of an acute complicated course in pediatric patients with Acute Hematogenous Osteomyelitis (AHO).

**Methods:**

A predictive model was developed based on a dataset of 82 pediatric AHO patients. Clinical data, imaging findings, and laboratory results were systematically collected for all patients. Subsequently, biomarker indices were calculated based on the laboratory results to facilitate a comprehensive evaluation. Univariate and multivariate logistic regression analyses were conducted to identify factors influencing early adverse outcomes in AHO. A nomogram model was constructed based on independent factors and validated internally through bootstrap methods. The discriminative ability, calibration, and clinical utility of the nomogram model were assessed using receiver operating characteristic (ROC) curves, calibration plots, and decision curve analysis (DCA), respectively. The developed nomogram model was compared with previously published A-score and Gouveia scoring systems.

**Results:**

Logistic regression analysis identified delayed source control, suppurative arthritis, albumin on admission, and platelet to lymphocyte ratio (PLR) as independent predictors of early adverse outcomes in pediatric AHO patients. The logistic regression model was formulated as: Log(P) = 7. 667–1.752 × delayed source control − 1.956 × suppurative arthritis − 0.154 × albumin on admission + 0.009 × PLR. The nomogram’s AUC obtained through Bootstrap validation was 0.829 (95% CI: 0.740–0.918). Calibration plots showed good agreement between predictions and observations. Decision curve analysis demonstrated that the model achieved net benefits across all threshold probabilities. The predictive efficacy of our nomogram model for acute complicated course in pediatric AHO patients surpassed that of the A-score and Gouveia scores.

**Conclusions:**

A predictive model for the acute complicated course of pediatric AHO was established based on four variables: delayed source control, suppurative arthritis, albumin on admission, and PLR. This model is practical, easy to use for clinicians, and can aid in guiding clinical treatment decisions.

## Background

Acute hematogenous osteomyelitis (AHO) in children represents a significant infectious disease challenge, with incidence rates estimated at 2–20 cases per 100,000 children in developed nations [1–4]. This rate is markedly higher in low- to middle-income countries, where the prevalence, pathogenic spectra, and severity of infections closely correlate with regional characteristics, predominantly involving *Staphylococcus aureus* [5, 6]. The advent of antimicrobial therapies has facilitated the effective management of most pediatric AHO cases through early empirical antibiotic intervention [7]. Traditionally, treatment for AHO relied on prolonged intravenous antimicrobial administration. However, recent evidence suggests that for cases with a less complicated trajectory, transitioning from short-term intravenous to oral antibiotic therapy ensures treatment efficacy and safety, reducing the need for extensive diagnostic procedures and invasive interventions, thereby significantly alleviating the treatment burden on patients and their families [8–11]. Nonetheless, for children at risk of adverse outcomes—potentially facing multiple surgeries, prolonged hospitalization, or even short-term recurrence—a long-term intravenous antibiotic regimen remains indispensable. Early assessment of disease severity to distinguish between children likely to develop unfavorable outcomes and those eligible for shorter courses of intravenous antibiotics is thus critically important.

Clinical, laboratory, and radiological parameters have traditionally been employed for risk stratification of patients with AHO to predict adverse outcomes. Notably, the Severity of Illness (SOI) scoring system introduced by Copley et al., along with the A-score and C-score assessments developed by Alhinai et al., have made significant contributions to this field [12, 13]. Recently, a model focusing on low inflammatory response, developed by researchers in Europe, represents an advancement in predictive model research, aiming to forecast bone joint infections and identify patients at low risk [14]. However, these models are in their nascent stages, with a glaring gap in external validation studies to affirm their scoring systems’ efficacy. Particularly in Asia, discrepancies in epidemiological characteristics, virulence, and antibiotic resistance among common AHO pathogens like *Staphylococcus aureus*, when compared to their counterparts in the United States and Europe, underscore the importance of developing and validating predictive models tailored to different geographical and clinical settings [15, 16].

Given the variability in clinical severity and treatment decision-making across regions, this study aims to develop a nomogram for predicting an acute complicated course in pediatric patients with AHO, tailored to a Chinese patient cohort.

## Materials and methods

### Patients

This study was approved by the ethics committee of our hospital (approval number 2021KS024). The requirement for informed patient consent was waived.

Patients diagnosed with AHO who were admitted for inpatient treatment at our hospital between January 2017 and January 2022 were retrospectively recruited for this study. All participant data were obtained retrospectively from their medical records. The specific criteria for inclusion and exclusion were as outlined below. Inclusion criteria for this study were: (1) a confirmed diagnosis of AHO; (2) presentation of symptoms for 14 days or less prior to hospital admission, coupled with the absence of antibiotic treatment prior to conducting blood tests; and (3) patient age ranging from 1 month to 18 years.Exclusion criteria encompassed: (1) patients with underlying conditions such as hematologic diseases, rheumatic diseases, cardiac diseases, malignant tumors, or chronic illnesses; (2) infections secondary to open fractures or surgical interventions; and (3) patients with incomplete medical records.

### Risk factors and definition of terms

Demographic information including, gender, age, site of infection, body temperature, culture results, number of surgeries performed, delayed source control, presence of purulent arthritis, complications, length of hospital stay, and MRI findings. Laboratory markers analyzed comprised erythrocyte sedimentation rate (ESR), white blood cell count (WBC), CRP levels, neutrophil count, lymphocyte count, monocyte count, platelet count, and serum albumin levels were extracted from the medical records. From these data, several ratios were calculated to provide further insights into the patients’ inflammatory and nutritional status, including the neutrophil to lymphocyte ratio (NLR), monocyte to lymphocyte ratio (MLR), platelet to lymphocyte ratio (PLR), C-reactive protein to albumin ratio (CAR), platelet to albumin Ratio (PAR), and the prognostic nutritional index (PNI). PNI was defined as albumin (g/L) + 5 *lymphocyte count (*10^9^/L).

The diagnostic criteria for AHO were established on two primary bases: the detection of pathogenic microorganisms in blood, pus, or tissue cultures represents the widely accepted standard. In cases where cultures were negative, an indirect diagnosis may be considered if clinical symptoms or signs of AHO such as fever, localized swelling, pain, or limited movement are present, alongside positive findings in C-reactive protein (CRP) levels, X-ray, or Magnetic Resonance Imaging (MRI), and an observable improvement within 48 h of initiating antibiotic therapy [17]. Surgical interventions in the management of AHO included bone debridement, abscess drainage, and osteoarticular incision and drainage. Delayed source control was defined as the initiation of the first surgical intervention after the third day of hospitalization. The term ‘multiple debridements’ referred to the surgical treatment of two or more non-adjacent bones. A disseminated infection was characterized by the development of multifocal infections, pneumonia, septic pulmonary embolism, deep vein thrombosis, or endocarditis during the hospital stay. Treatment failure was identified by the persistence of symptoms and/or signs that lead to readmission or a change in antibiotic treatment within six weeks of the initial treatment, despite apparent adherence to antibiotic protocols. An acute complicated course of AHO was defined by any of the following criteria: ≥2 surgical interventions, hospitalization for more than 21 days, or treatment failure.

### Statistical analysis

Data analysis was conducted utilizing SPSS software, version 26.0. Quantitative data, adhering to a normal distribution, were expressed as mean ± standard deviation, while those not conforming to normal distribution were presented as median (P25, P75). Categorical data were represented by frequency and proportion. Independent sample t-tests were employed for normally distributed quantitative data, whereas non-parametric tests were utilized for data not following a normal distribution. Chi-square tests were applied for categorical data.

A univariate analysis was performed on all variables, with statistically significant factors included in a stepwise backward multiple logistic regression model. Subsequently, the independent predictors identified were incorporated into a nomogram model using R software, version 4.2.2, aiming to predict the early adverse outcomes in pediatric AHO. The predictive performance of the nomogram was assessed through internal validation via bootstrap resampling (1000 iterations), constructing receiver operating characteristic (ROC) curves. The fit between predicted and observed outcomes was evaluated using the Hosmer-Lemeshow test, while calibration was assessed via calibration curves. Decision curve analysis was conducted to examine the net benefit of the nomogram at various threshold probabilities, thereby evaluating its predictive capacity. A *p*-value of < 0.05 was considered statistically significant. Additionally, the cohort data were subjected to risk prediction using the A-score [13] and Gouveia score [14], facilitating a comparison of effectiveness with the nomogram model developed in this study.

## Results

**Fig. 1:**
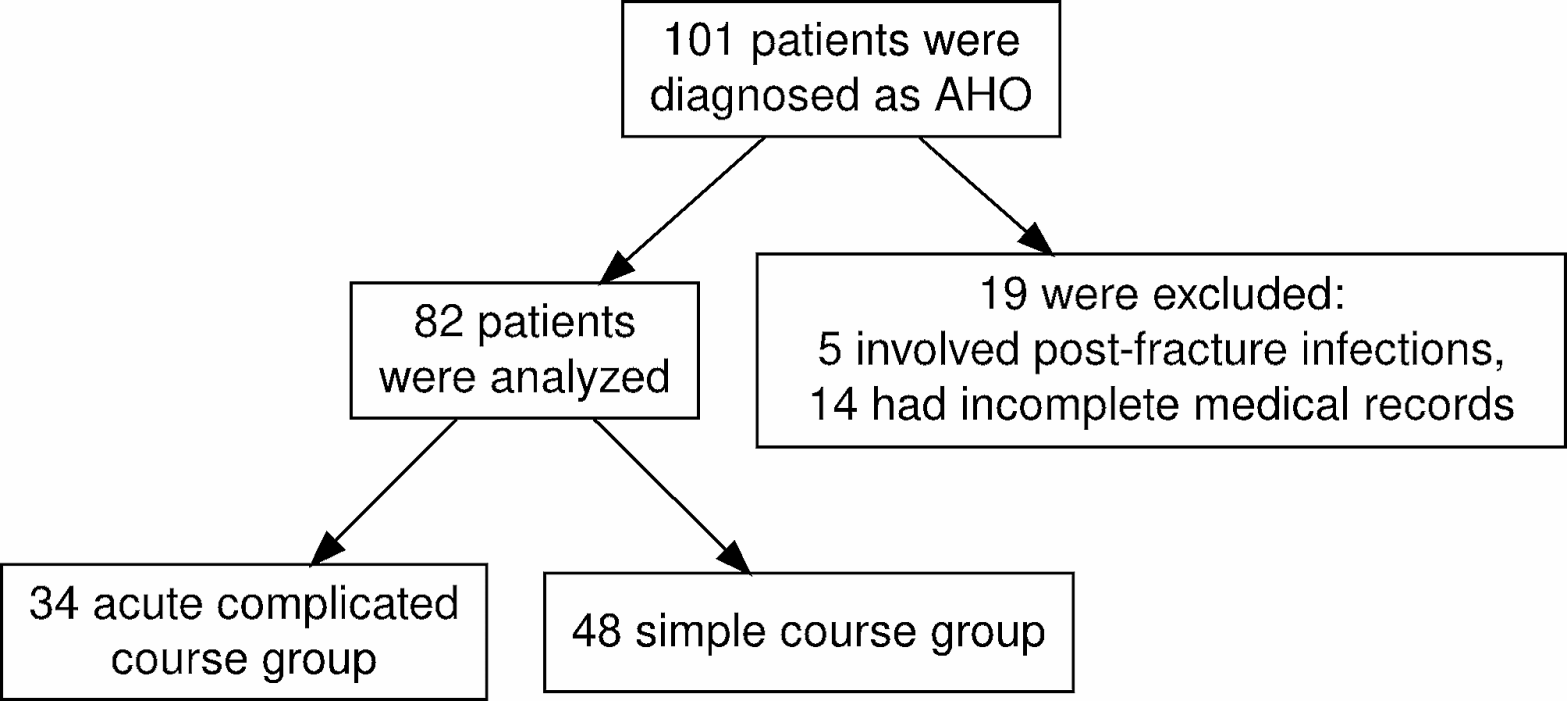
The flow chart of the study

In total, 101 patients were diagnosed with AHO. Of these, 19 were excluded from the study for the following reasons: 5 cases involved post-fracture infections, and 14 had incomplete medical records. The latter group included 11 patients who were transferred to our department after receiving initial treatment in other hospitals or departments, and 3 who discontinued their treatment and self-discharged. As a result, 82 patients were ultimately enrolled in the study. The flowchart is shown in Fig. 1.

### General information

82 patients with AHO were included in this study, the gender distribution comprised 47 males and 35 females, with a median age at presentation of 7.2 years. Upon admission, 37 patients (45.1%) exhibited fever as a symptom, and 30 (36.6%) continued to exhibit fever despite 48 h of empirical intravenous antibiotic treatment. The tibia was the most commonly involved site (23 cases, 28.1%), followed by the femur (20 cases, 24.5%), humerus (12 cases, 14.6%), calcaneus (8 cases, 9.8%), fibula (6 cases, 7.3%), pelvis (5 cases, 6.1%), patella (2 cases, 2.4%), radius (2 cases, 2.4%), clavicle (1 case, 1.2%), vertebra (1 case, 1.2%), fingers (1 case, 1.2%), and toes (1 case, 1.2%). Concomitant Suppurative arthritis was identified in 12 patients (14.6%).

Bacterial cultures were performed for 70 patients, yielding a blood culture positivity rate of 19.7% (12/61) and a positivity rate of 81.8% (27/33) for cultures from pus, synovial fluid, or tissue. Among the 33 cases with positive microbial cultures, there was one case each of *Salmonella enterica* and *Klebsiella pneumoniae* (each comprising 3% of positive cases), while *Staphylococcus aureus* was identified in 31 cases (94%), of which 22 were methicillin-sensitive *Staphylococcus aureus* (MSSA) and 9 were methicillin-resistant *Staphylococcus aureus* (MRSA). Moreover, MRI of the suspected infection sites was conducted in 71 patients (86.6%), revealing bone abscess in 39 cases (47.6%) and multifocal infections in 3 cases (3.7%).

### Univariate logistic regression analysis

In the acute phase of the study, patients were stratified into two groups based on the severity of their condition: those with an acute complicated course (*n* = 34) and those with a simple course (*n* = 48). Univariate analysis revealed significant associations between the development of an acute complicated course in pediatric patients with AHO and several factors: bone abscess, delayed source control, suppurative arthritis, surgical interventions, ESR on admission, CRP peak, CRP ≥ 100 mg/L after 2–4 d of antibiotics, albumin on admission, PLR, CAR, PAR, and PNI (*p* < 0.05 for all) (Table 1).

**Table 1:** Univariate logistic regression analysis of various risk factors

Variables	Total cases(*n* = 82)	Acute complicated course(*n* = 34)	Simple course(*n* = 48)	*P*
Age, years, median(IQR)	7.2 (2.5,10.1)	7.1 (1.5,10.8)	7.3(3.2,9.9)	0.608
Gender, *n* (%)				0.825
Male	47(57.3)	19(55.9)	28(58.3)	——
Female	35(42.7)	15(44.1)	20(41.7)	——
Fever at admission, *n* (%)	37(45.1)	18(39.6)	19(52.9)	0.231
Fever > 48 h of antibiotics, *n* (%)	30(36.6)	16(47.1)	14(29.2)	0.097
Multifocal infection, *n* (%)	3(3.7)	2(5.9)	1(2.3)	0.367
Bone abscess, *n* (%)	39(47.6)	22(64.7)	17(35.4)	0.009
MRSA, *n* (%)	9(11.0)	6(17.6)	3(6.3)	0.104
Staphylococcus aureus, *n* (%)	31(37.8)	17(50)	14(29.2)	0.055
Delayed source control, *n* (%)	21(25.6)	16(47.1)	5(10.4)	< 0.001
Suppurative arthritis, *n* (%)	12(14.6)	9(26.5)	3(6.3)	0.011
Surgical interventions, *n* (%)	38(46.3)	24(70.6)	14(29.2)	< 0.001
WBC count, ×10^9^/L, median (IQR)	11.9 (8.6,14.0)	11.9(8.8,13.8)	11.9 (8.3,14.3)	0.891
ESR on admission, mm/L, median (IQR)	40 (23.0,62.0)	53.5(36.0,70.0)	29 (18.5,52.5)	0.003
CRP on admission, mg/L, median (IQR)	55.6 (23.2,99.8)	67.1(28.3,106.0)	39.9 (15.5,77.1)	0.06
CRP peak, mg/L, median (IQR)	67.6 (25.2,117.4)	106.8(53.6,163.2)	46.0 (14.6,82.1)	0.002
CRP ≥ 100 mg/L after 2–4 d of antibiotics, *n* (%)	11(13.4)	2(4.2)	9(26.5)	0.004
Albumin on admission, g/L, median (IQR)	42.5 (38.9,44.4)	40.7(37.4,43.5)	42.8 (41.2,44.6)	0.017
Neutrophil count, ×10^9^/L, median (IQR)	7.5 (4.5,9.9)	7.5(4.9,10.0)	7.3 (3.7,9.5)	0.449
Lymphocyte count, ×10^9^/L, median (IQR)	2.7 (1.5,4.4)	2.3(1.4,4.1)	2.9 (1.8,4.6)	0.153
Monocyte count, ×10^9^/L, median (IQR)	0.9 (0.6,1.3)	0.9(0.6,1.3)	0.9 (0.6,1.2)	0.645
Platelet count, ×10^9^/L, median (IQR)	319 (261,412)	346(270,430)	306 (244,395)	0.211
NLR, median (IQR)	2.6 (1.5,5.0)	2.6(1.9,5.5)	2.2 (1.0,4.9)	0.169
MLR, median (IQR)	0.4 (0.2,0.6)	0.4(0.2,0.6)	0.3 (0.2,0.5)	0.095
PLR, median (IQR)	119.6 (90.3,182.0)	145.4(114.4,236.4)	101.2 (80.8,145.8)	0.003
CAR, median (IQR)	1.3 (0.5,2.7)	1.8(0.7,3.2)	1.0 (0.4,2.0)	0.023
PAR, median (IQR)	7.8 (6.1,9.7)	8.6(6.7,10.9)	7.0 (5.4,9.1)	0.042
PNI, median (IQR)	56.5 (48.2,63.3)	53.9(46.7,61.2)	57.6 (51.6,67.5)	0.025

MRSA, methicillin-resistant Staphylococcus aureus; WBC, white blood cell count; ESR, Erythrocyte sedimentation rate; CRP, C-reactive protein; NLR, Neutrophil to lymphocyte ratio; MLR, Monocyte to lymphocyte ratio; PLR, Platelet to lymphocyte ratio; CAR, C-reactive protein to albumin ratio; PAR, Platelet to albumin ratio; PNI, prognostic nutritional index

### Multivariate regression analysis and the development of a predictive model

**Fig. 2:**
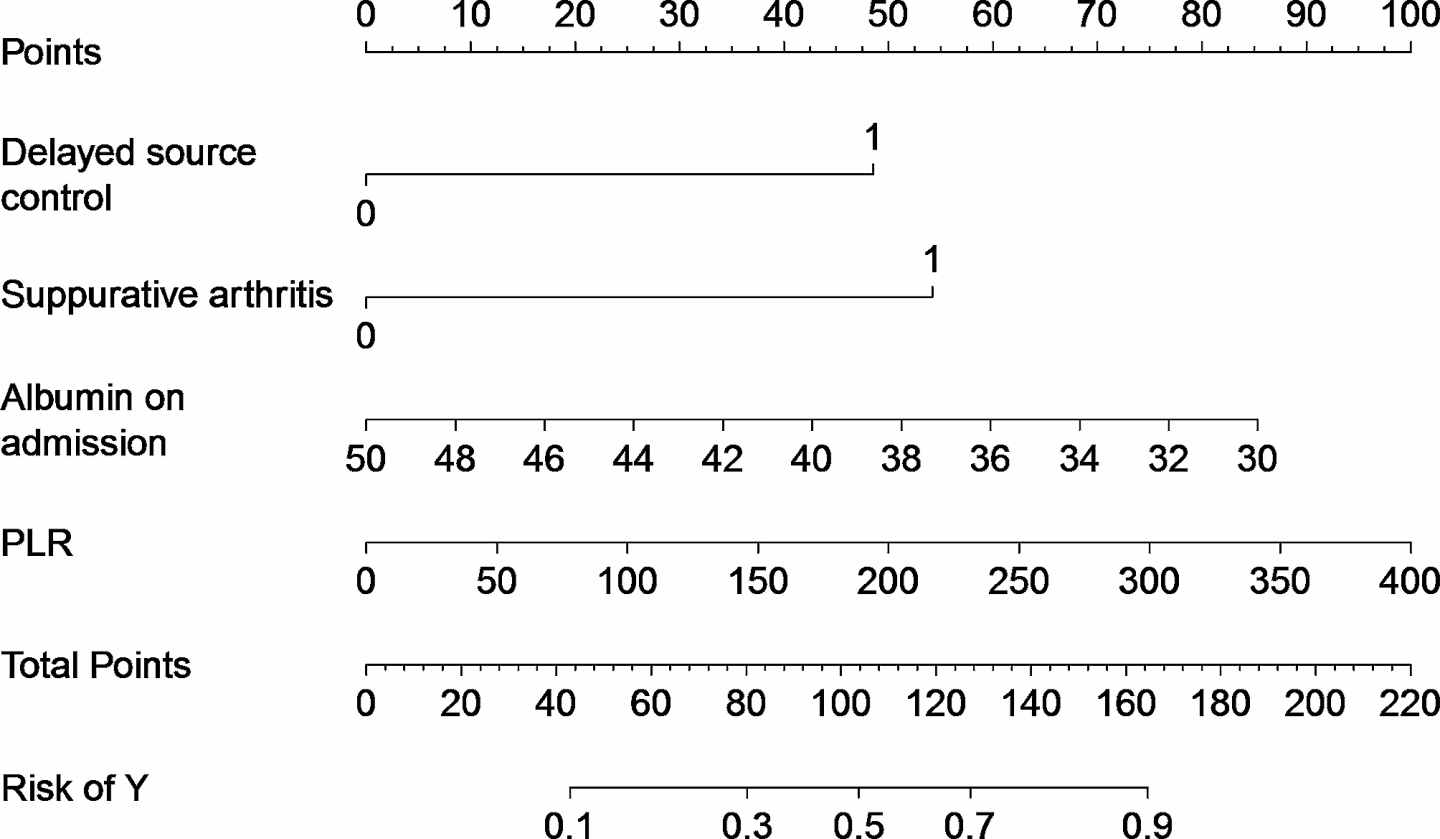
The nomograms for predicting the risk of acute complicated course in pediatric patients with AHO

Subsequent multivariate analysis, incorporating the 12 statistically significant variables from the univariate analysis, employed a backward stepwise logistic regression approach. This analysis identified delayed source control, suppurative arthritis, albumin on admission, and PLR as independent predictors for the occurrence of an acute complicated course in AHO patients (*p* < 0.05 for each). The logistic regression model derived from these predictors is expressed as: Log(P) = 7.667–1.752 × delayed source control − 1.956 × suppurative arthritis − 0.154 × albumin on admission + 0.009 × PLR (Table 2). A nomogram was constructed based on this logistic regression equation to facilitate the prediction of an acute complicated course (Fig. 2). The public online version of our nomogram is available at https://zcczy712.shinyapps.io/dynnomapp/.

**Table 2:** Binary logistic regression analysis backwards stepwise (conditional) for acute complicated course

Variables	*B*	Standard Error	c²	*P*	χ^2^	95% CI for Odds Ratio Lower Upper	
Delayed source control	-1.752	0.649	7.274	0.007	0.173	0.049	0.62
Suppurative arthritis	-1.956	0.833	5.514	0.019	0.141	0.028	0.724
Albumin on admission	-0.154	0.071	4.762	0.029	0.857	0.746	0.984
PLR	0.009	0.003	6.926	0.008	1.009	1.002	1.016
Intercept	7.667	3.066	6.254	0.012	2137.061	——	

PLR, Platelet to lymphocyte ratio

### Validation of the prognostic nomogram

**Fig. 3:**
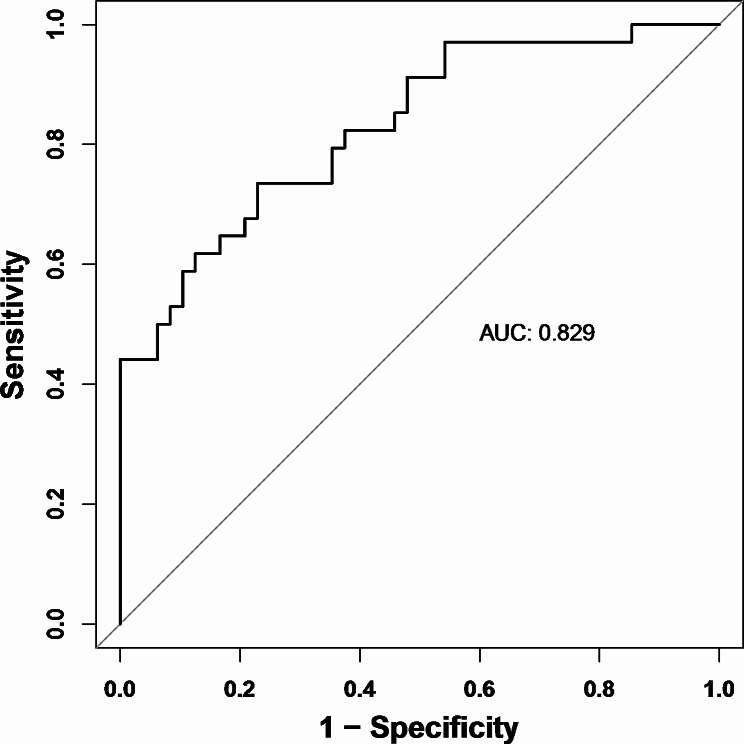
ROC for the risk of acute complicated course in AHO patients. ROC, receiver operating characteristic; AUC, area under the curve

In the internal validation of the nomogram for predicting acute complicated course in pediatric patients with AHO, bootstrap resampling (*n* = 1000 iterations) was employed. This validation process revealed an Area Under the Curve (AUC) of 0.829 (95%CI: 0.740–0.918), indicating a high predictive accuracy. The optimal cutoff value, identified at 0.44 where the sum of sensitivity and specificity peaked, resulted in a sensitivity of 73.5% and a specificity of 77.1%. The model demonstrated a positive predictive value (PPV) of 69.4%, a negative predictive value (NPV) of 80.4%, and an overall accuracy of 75.6%, thus exhibiting good discriminative ability (Fig. 3; Table 3).

**Table 3:** Comparative ROC curve analysis for acute complicated course in pediatric AHO patients

Variables	AUC	Cutoff value	Sensitivity	Specificity	PPV	NPV	Youden index	Accuracy
Nomogram	0.829	0.44	0.735	0.771	0.694	0.804	0.506	0.756
A-score	0.777	4	0.735	0.729	0.658	0.795	0.464	0.732
Gouveia score	0.689	0.2	0.882	0.437	0.526	0.84	0.319	0.622

AUC, area under the curve; PPV indicates positive predictive value; NPV, negative predictive value

**Fig. 4:**
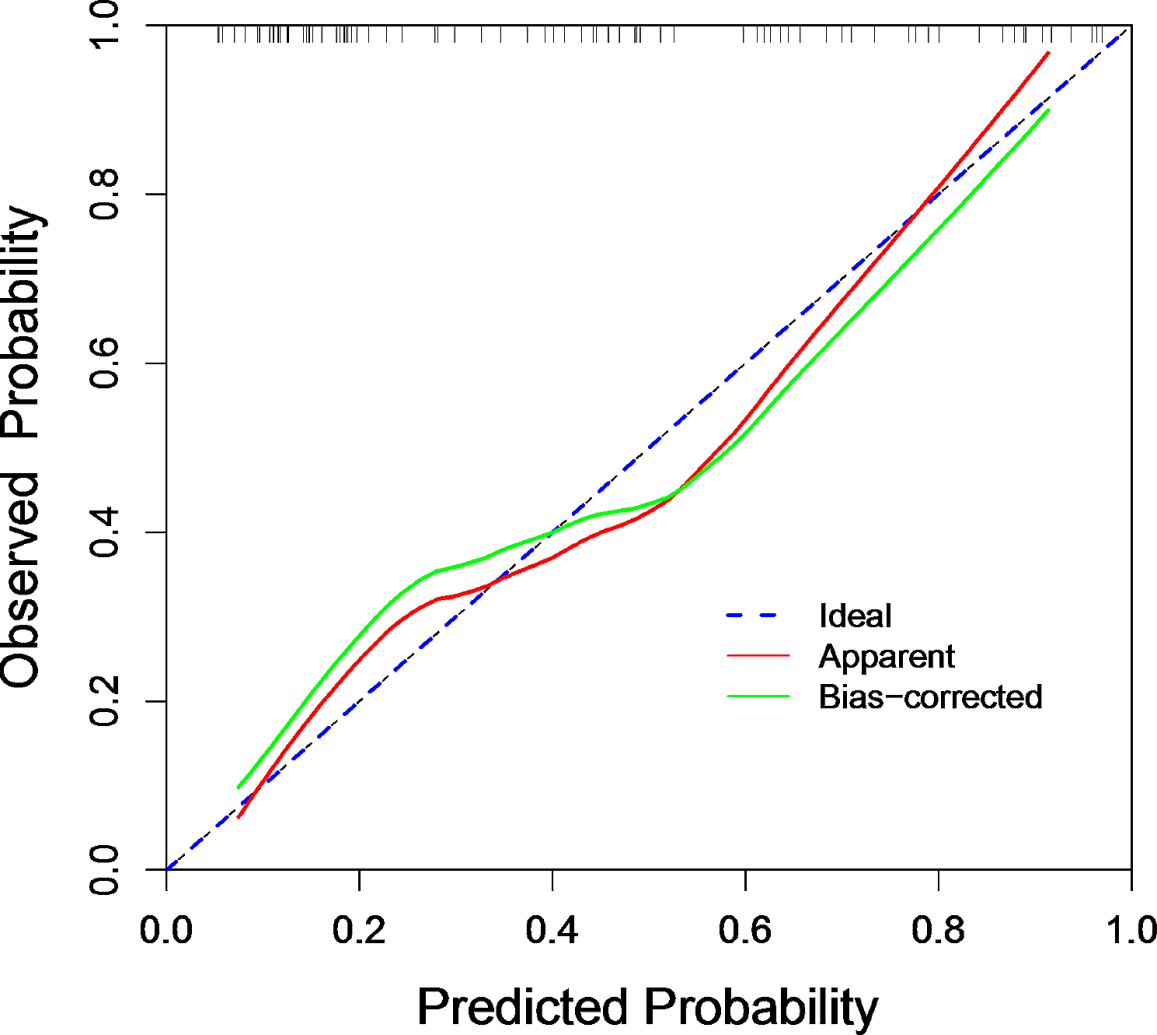
Calibration curves of the nomogram prediction. The x-axis and the y-axis represent the nomogram prediction and the actual situation, respectively. The diagonal line shows that the forecast is exactly what happened. The more the solid line matches the diagonal, the better the predictive accuracy

**Fig. 5:**
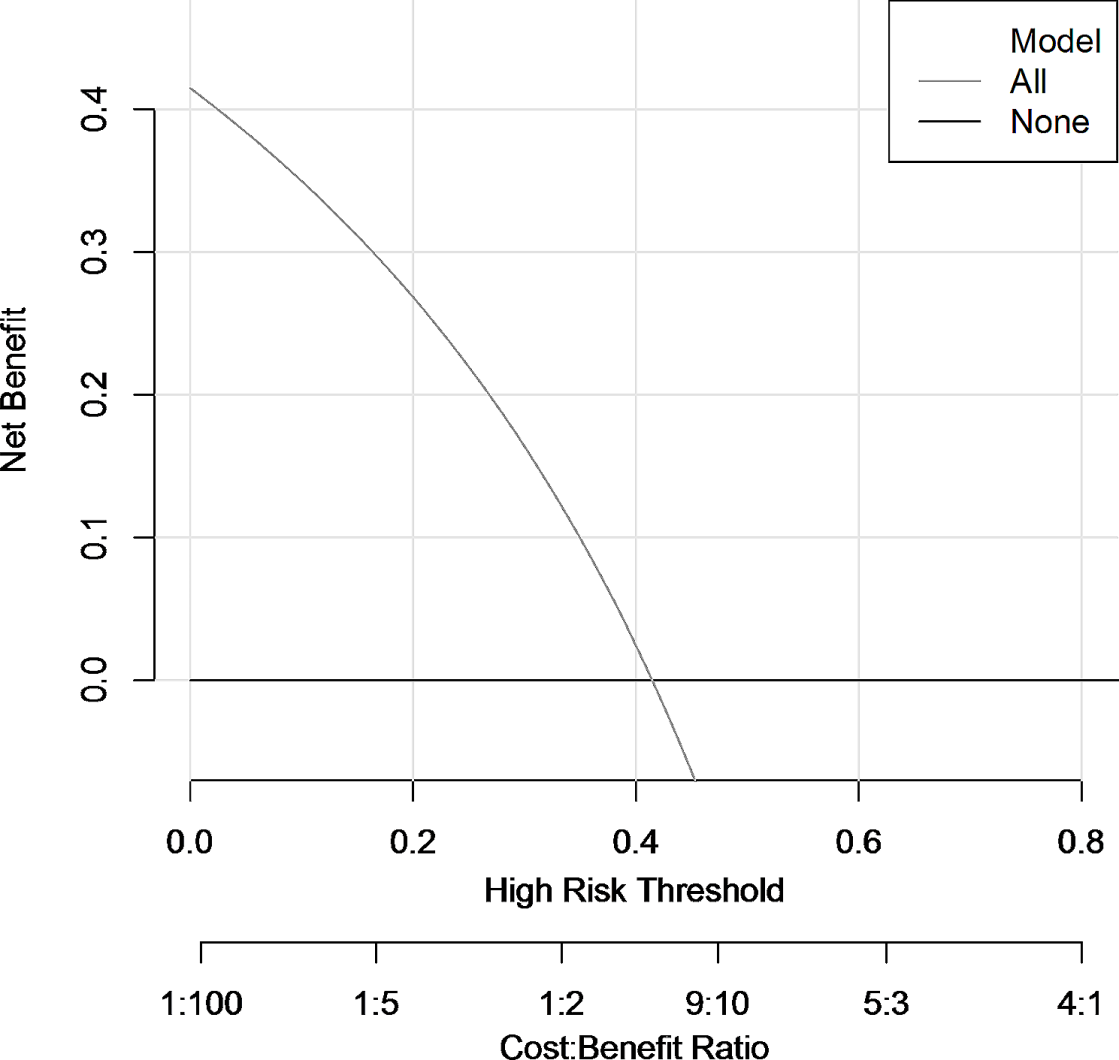
Decision curve analysis (DCA) for the predictive model. The net benefit was produced against the high-risk threshold. The red line represents the predictive model. The application of this predictive model would add net benefit compared with either the treat-all or the treat-none strategies

The calibration curve, comparing predicted against observed outcomes, confirmed the model’s reliability in the validation set (Fig. 4). Additionally, the Hosmer-Lemeshow test yielded a *p*-value of 0.27, suggesting a non-significant deviation from perfect model fit, further affirming the model’s calibration. The decision curve analysis (DCA) underscored the model’s clinical utility, indicating a net benefit across a range of threshold probabilities for predicting adverse outcomes in AHO patients (Fig. 5).

In a comparative analysis, the predictive model for acute complicated course in pediatric patients with AHO was juxtaposed with the A-score and Gouveia scoring systems (Table 3). The A-score, when applied to the cohort for predicting the risk of early adverse outcomes in AHO patients, yielded an AUC of 0.777 (95%CI: 0.673–0.882). With a designated cutoff value of 4, the A-score demonstrated a sensitivity of 73.5% and a specificity of 72.9%, alongside a PPV of 65.8%, a NPV of 79.5%, and an overall accuracy of 73.2%. In comparison to our model, while the sensitivity remained similar, the A-score displayed lower specificity, PPV, NPV, and accuracy.

Conversely, the application of the Gouveia score yielded an AUC of 0.689 (95%CI: 0.575–0.804), indicating moderate predictive performance. At a cutoff value of 0.2, the Gouveia score exhibited a sensitivity of 88.2% and a specificity of 43.7%, with a PPV of 52.6% and an NPV of 84.0%, culminating in an accuracy of 62.2%. This suggests that, compared to our nomogram, both the A-score and Gouveia scoring system demonstrate lower predictive efficacies, with the Gouveia score in particular showing a markedly lower specificity and overall accuracy.

## Discussion

Currently, clinical prognostication of adverse outcomes in pediatric AHO utilizes a variety of scoring systems, including the SOI score, A-score, C-score, and the Gouveia score, as documented in the literature [12–14]. Notably, while these scoring systems play a pivotal role in clinical practice, the incorporation and thorough assessment of novel serum biomarkers in predicting the acute complicated course of AHO in children have not been extensively explored. Therefore, this study endeavors to integrate novel serum biomarkers as observational indicators for the early prediction of adverse prognostic outcomes, aiming to furnish more precise guidance for the early assessment of disease severity.

In our study, conducted within a Chinese demographic, we innovatively utilized serum biomarkers as observational indicators. The demographic and clinical profile of our cohort, including median age, gender distribution, and the causative organisms of AHO, aligned with the characteristics reported in prior studies on AHO [18]. Univariate analysis revealed that novel biomarkers—such as albumin on admission, PLR, CAR, PAR, and PNI, —were potentially associated with the development of an acute complicated course in pediatric AHO. Subsequent multivariate analysis identified delayed source control, suppurative arthritis, albumin on admission, and PLR as independent risk factors for predicting an acute complicated course in children with AHO. Based on these findings, a nomogram was constructed for visual representation, enhancing the predictive model’s accessibility and interpretability.

The correlation between delayed source control and the presence of concurrent suppurative arthritis with adverse outcomes in pediatric AHO has been corroborated by previous research [19]. Alhinai et al. proposed the A-score system, which consists of five components: bone abscess, fever after 48 h of starting antibiotics, suppurative arthritis, disseminated disease, and delayed source control. The A-score evaluates the severity by assigning cumulative points to each factor, notably assigning higher weights to concurrent Suppurative arthritis and delayed source control, with 3 and 4 points respectively, underlining the critical importance of these two factors. Our study’s nomogram model, which incorporates delayed surgical intervention and concurrent Suppurative arthritis as independent predictive factors, aligns with variables of the A-score, thereby reinforcing the consensus recommendations for the treatment of pediatric AHO. Specifically, the consensus advocates for assessing the necessity of surgical intervention when AHO infections progress rapidly or when there is no significant clinical improvement after 2–4 days of antimicrobial treatment [17].

Nutritional status plays a pivotal role in maintaining health and preventing infections [20]. Our study corroborates the notion that serum albumin levels upon admission serve as an independent prognostic factor for the development of acute complicated courses in patients with AHO. Despite limited research specifically addressing serum albumin in pediatric AHO, its role in inflammation modulation is well-documented. Yuwen et al. observed that orthopedic patients with serum albumin below 35 g/L face a 2.5 times higher risk of postoperative infections [21]. Moreover, Holmes et al. linked low serum albumin levels to increased treatment failures in *Staphylococcus aureus* bacteremia within 30 days [22]. Integrating serum albumin levels with CRP levels, platelet counts, and lymphocyte counts facilitates the calculation of CAR, PAR, and PNI. These metrics collectively offer insights into the patient’s nutritional and immune status, thereby presenting novel perspectives for assessment. Ren et al. [23] have suggested that a CAR value exceeding 0.447 at admission is an independent predictor for pediatric septic arthritis. Concurrently, Li et al. [24] found that PNI levels were inversely correlated with the inflammatory marker CRP and duration of hospital stay in neonatal sepsis. Univariate logistic regression analysis in our study indicated that serum albumin, CAR, PAR, and PNI could all serve as predictors for the progression to acute complicated courses in pediatric AHO. However, multivariate logistic regression analysis revealed that only serum albumin levels at admission were independent predictors of such outcomes. This underscores the central role of nutritional status in predicting the early adverse prognosis in AHO.

PLR is increasingly recognized as a predictive marker of inflammatory processes. In the context of exploring PLR as a biomarker for disease severity and prognosis, a multicenter retrospective study conducted by Simon et al. [25] has provided compelling evidence of its significance. Notably, within COVID-19 case studies, elevated PLR values were significantly associated with an increased risk of mortality. Building on the findings of Simon et al., research by Asperges and colleagues further underscored the correlation between PLR and both the severity of COVID-19 and mortality rates, offering additional support for the utility of PLR as a prognostic marker [26]. Moreover, the application of PLR in predicting outcomes in patients with acute kidney injury showcases its promising utility, highlighting its potential importance in clinical prognostic assessments [27]. Our study findings indicate that PLR serves as an independent factor predicting the development of acute complicated courses in pediatric AHO. There is reason to believe that PLR levels may correlate with the severity of disease in children with AHO. However, this conclusion warrants further confirmation through studies with larger sample sizes.

Our research identifies critical risk factors influencing the clinical manifestations, laboratory assessments, and MRI findings in AHO. Nonetheless, it is crucial to also consider additional risk factors such as impairments in skin integrity and vascular insufficiency, which substantially influence AHO risk, especially in primary care settings. Given the practical limitations of MRI in these settings, alternatives like ultrasound and plain radiography become essential, particularly where MRI access is restricted. These observations underline the necessity for a comprehensive approach in evaluating AHO risk factors and diagnostic strategies within primary healthcare frameworks.

Considering the ease of access to risk variables, this study opted to compare the effectiveness of our nomogram model with the A-score and Gouveia scoring systems. While these scoring systems have exhibited satisfactory predictive performance within their respective local cohorts, our findings suggest that, particularly for patients in the Chinese region, our nomogram model provides superior accuracy in predicting the severity of pediatric AHO. It can assist clinicians in identifying patients with a lower risk of developing early adverse outcomes, thereby guiding them to switch from intravenous to oral antibiotic therapy in the early stages. For patients at higher risk, more aggressive treatment strategies should be adopted. Nonetheless, there remains room for further optimization.

This study has several limitations. Firstly, the data for this study were derived from patients with AHO in China, as part of a single-center retrospective analysis, which poses a potential for information bias that may affect the generalizability of the results. Secondly, the sample size of this study was small, precluding the establishment of a model for predicting long-term complications, and lacked an external dataset for validation. Future studies will require multicenter, large-sample clinical data to refine this predictive model, as well as to develop models for predicting late adverse outcomes.

## Conclusion

Our prediction model has demonstrated certain applicability in predicting the risk of children with AHO developing an acute complicated course of the disease. Future research is needed to further validate the effectiveness and wide applicability of this predictive model.

## Data Availability

The original contributions presented in the study are included in the article, further inquiries can be directed to the corresponding author.

## Abbreviations

AHO: Acute Hematogenous Osteomyelitis
ROC: Receiver operating characteristic
DCA: Decision curve analysis
PLR: Platelet to lymphocyte ratio
SOI: Severity of Illness
CRP: C-reactive protein
ESR: Erythrocyte sedimentation rate
WBC: White blood cell
NLR: Neutrophil to lymphocyte ratio
MLR: Monocyte to lymphocyte ratio
CAR: C-reactive protein to albumin ratio
PAR: Platelet to albumin Ratio
PNI: Prognostic nutritional index
MSSA: Methicillin-sensitive Staphylococcus aureus
MRSA: Methicillin-resistant Staphylococcus aureus
MRI: Magnetic Resonance Imaging
AUC: Area Under the Curve
PPV: Positive predictive value
NPV: Negative predictive value
DCA: Decision curve analysis
